# Supplementary figures and images for: A kinase-independent function of PAK is crucial for pathogen-mediated actin remodelling
Source: PLoS Pathog. 2021 Aug 30;17(8):e1009902. doi: 10.1371/journal.ppat.1009902 (PMC8432889; doi:10.1371/journal.ppat.1009902)

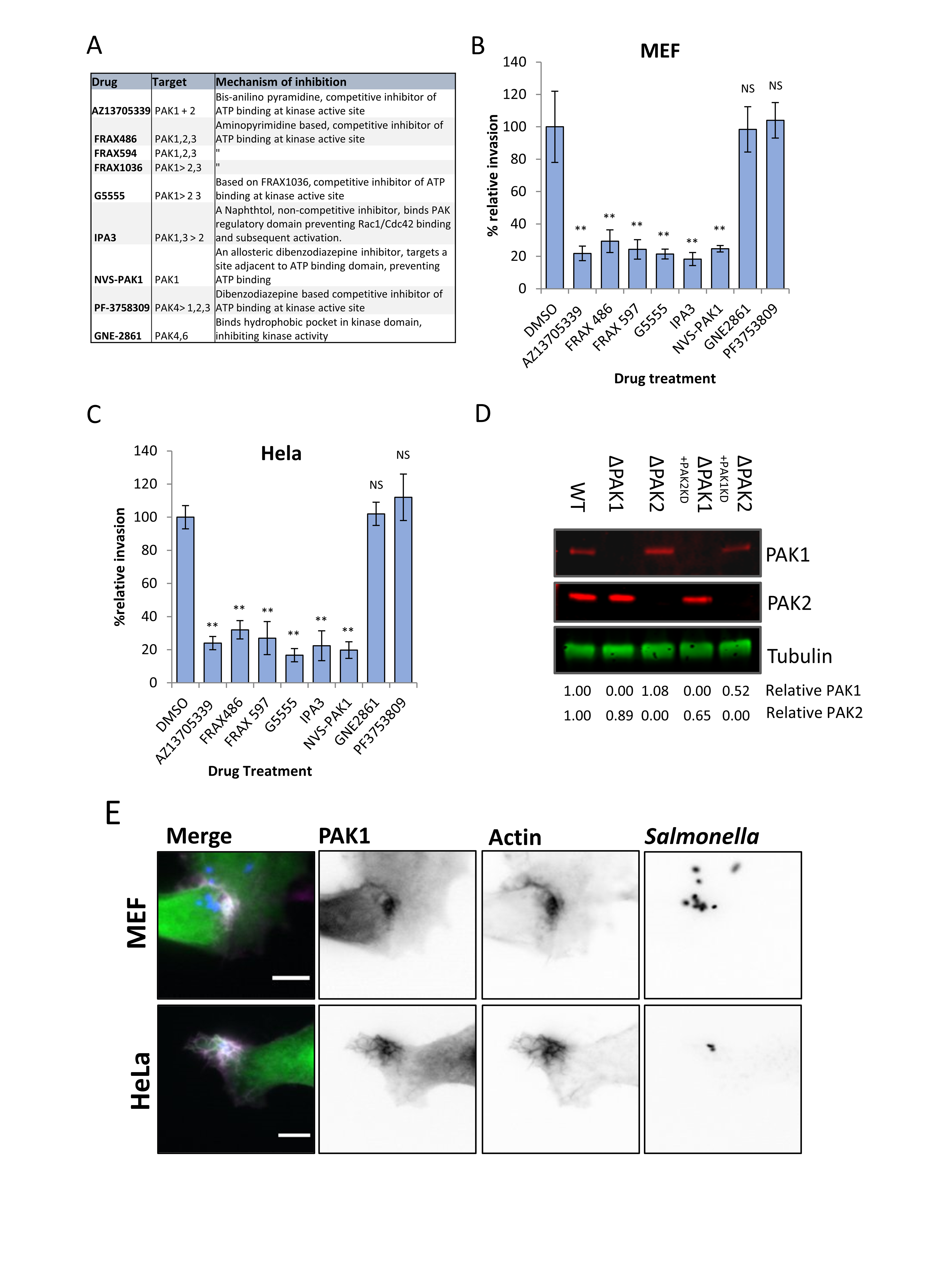

Supplement: S1 Fig — (A) Summary of drugs used and their mechanisms of action. Invasion of WT Salmonella (20 mins) into (B) WT MEF cells or (C) WT HeLa cells, each either pretreated with DMSO (control) or one of the PAK inhibitors AZ13705339, FRAX 486, FRAX 597, FRAX 1036, G5555, IPA3, NVS-PAK1, GNE-2861 or PF3758309. Invasion values are relative to those in control DMSO treated MEFs or HeLas respectively. (D) Immunoblot of cell lysates depicting relative amounts of PAK1 and PAK2 in WT, ΔPAK1, ΔPAK2 as well as ΔPAK1 cells pretreated with PAK1 siRNA, and ΔPAK2 cells pretreated with PAK2 siRNA. Levels of PAK1 and PAK2, quantified from band intensities and normalised to total tubulin levels, are displayed at the bottom. (E) Fluorescence microscopy of MEF and HeLa cells expressing Emerald-PAK1 (green), infected with WT Salmonella (10 mins) that had been pre-stained with Alexa-Fluor 350 (blue). Cells also stained with Texas-Red Phalloidin to visualise actin (purple). Scale bar is 10 μm. All Error bars indicate SD. NS–no significant difference, **—P <0.01, * P <0.05 (ANOVA followed by a post hoc Dunnett’s comparison). (TIF) [file ppat.1009902.s001.tif]

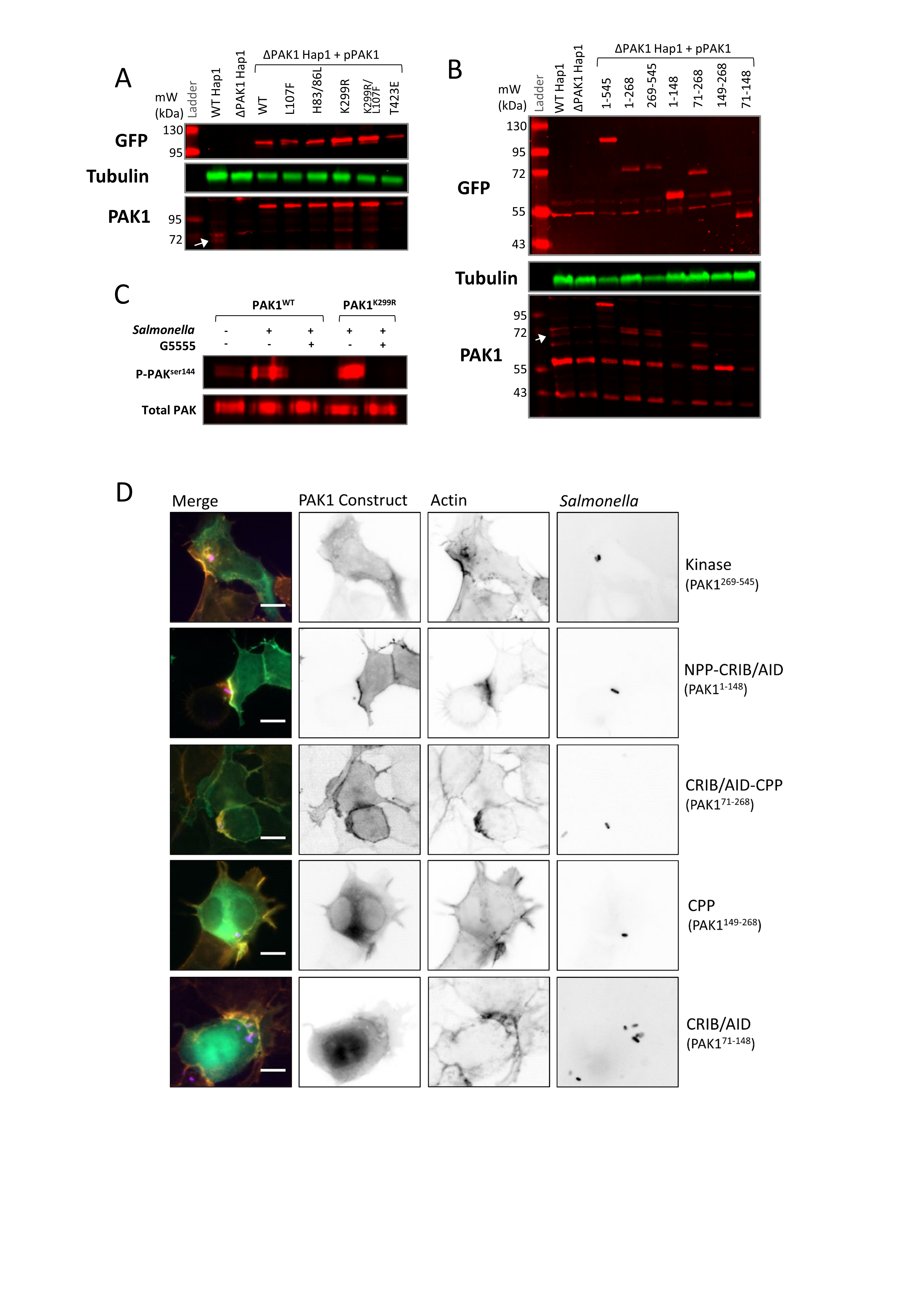

Supplement: S2 Fig — Expression of Full length (A) or truncated (B) PAK constructs confirmed by immunoblotting of corresponding cell lysates using a GFP antibody (Red), and a tubulin antibody (Green) as a loading control. Endogenous detected using a PAK1 antibody. (C) Immunoblot of total (Total PAK) and active (P-PAK1Ser144) Emerald-PAK1 or Emerald PAK1K299R, isolated from transfected ΔPAK1 Hap1 cells using a GFP-Trap. Cells were serum starved overnight, then either left uninfected, or infected with WT Salmonella (20 mins) in the absence or presence of G5555. (D) Fluorescence microscopy of ΔPAK1 Hap1 cells expressing emerald-tagged PAK constructs indicated (green), infected (10 mins) with Alexa Fluor 350-stained WT Salmonella (blue). Actin stained with Texas-Red Phalloidin (red). Scale bar is 10 μm. (TIF) [file ppat.1009902.s002.tif]

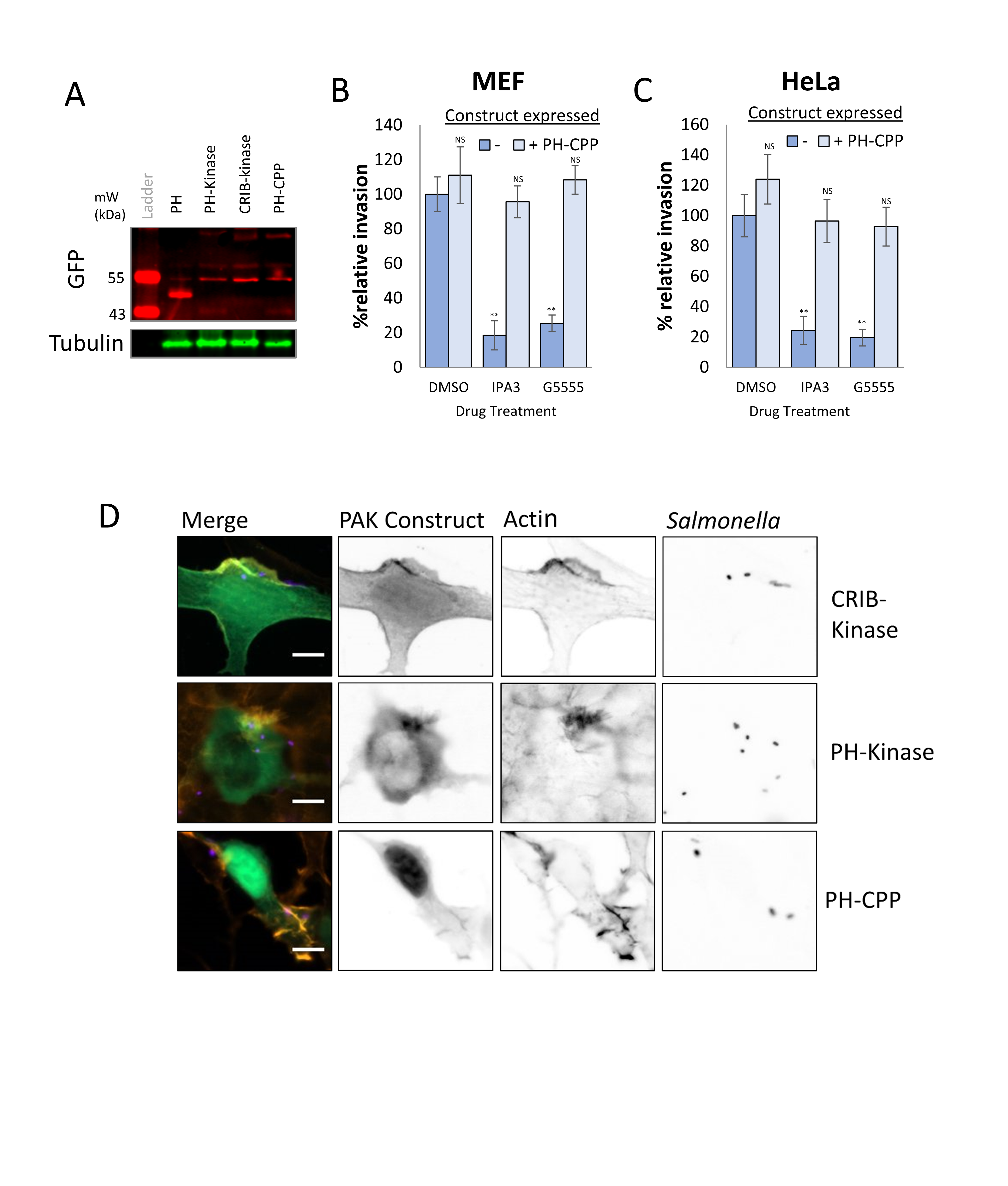

Supplement: S3 Fig — (A) Expression of constructs (PH, PH-kinase, CRIB-kinase and PH-CPP) confirmed by immunoblotting of corresponding cell lysates using a GFP antibody (Red), and a Tubulin antibody (Green) as a loading control. Invasion of WT Salmonella (20 mins) into (B) WT MEF cells or (C) WT HeLa cells, each either pretreated with DMSO (control), IPA3 or G5555, cells expressed either no construct (-) or PH-CPP. Invasion values are relative to those in control DMSO treated MEFs or HeLas respectively (D) Fluorescence microscopy of ΔPAK1 Hap1 cells expressing the Emerald-tagged PAK1 constructs indicated (green), infected (10 mins) with Alexa Fluor 350-stained WT Salmonella (blue). Actin stained with Texas-Red Phalloidin (red). Scale bar is 10 μm. All Error bars indicate SD. NS–no significant difference, **—P <0.01, * P <0.05 (ANOVA followed by a post hoc Dunnett’s comparison). (TIF) [file ppat.1009902.s003.tif]

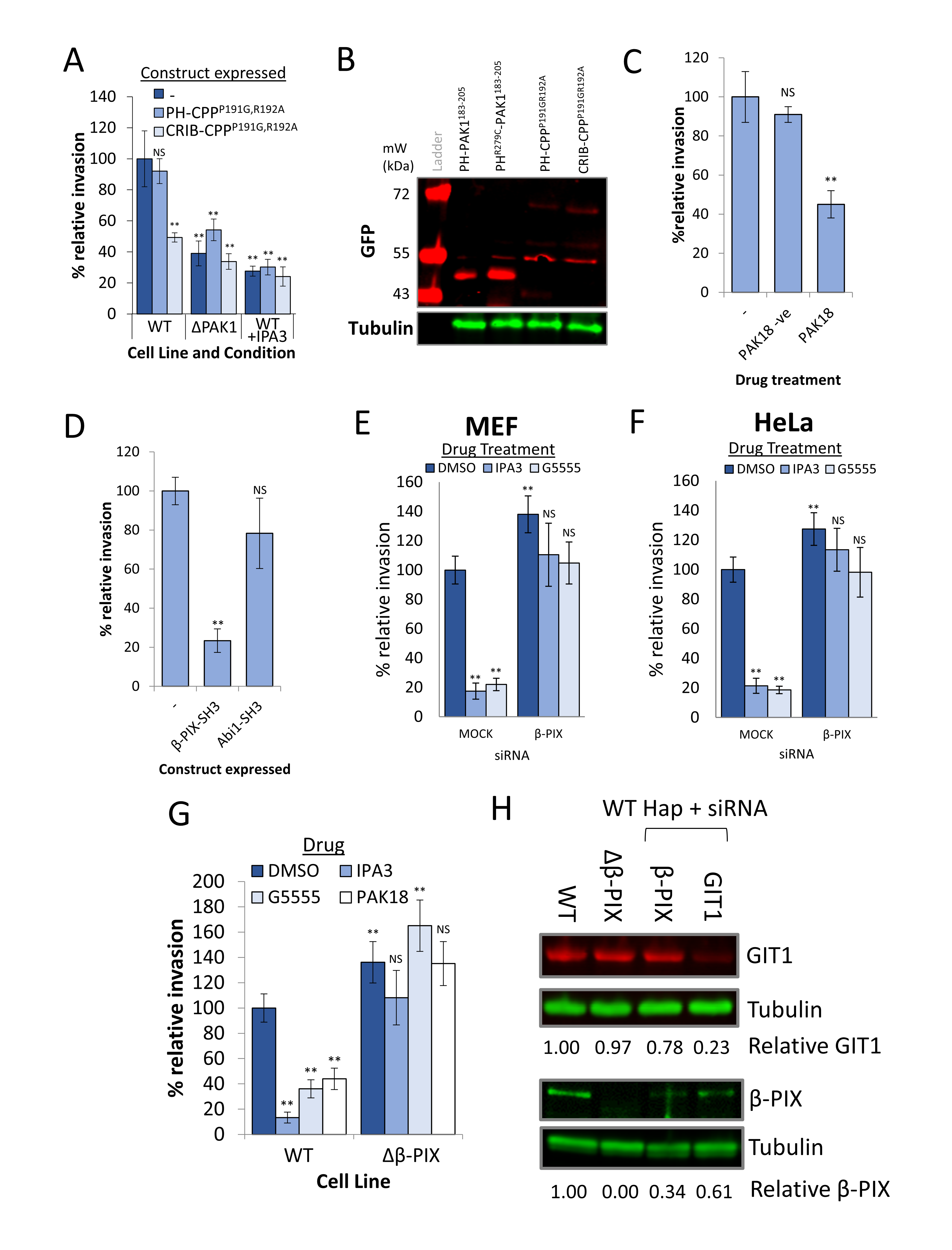

Supplement: S4 Fig — (A) Invasion of WT Salmonella into WT, ΔPAK1 or WT Hap1 cells pretreated with IPA3. Cells expressed the indicated construct, as described in (Fig 4A) (B) Expression of constructs indicated confirmed by immunoblotting of corresponding cell lysates using a GFP antibody (Red), and a Tubulin antibody (Green) as a loading control. (C) Invasion of WT Salmonella into WT Hap1 cells treated with control DMSO (-), PAK inhibitor PAK18 or the negative control version of PAK18 (PAK18 -ve). Invasion values are relative to those in control DMSO-treated cells. (D) Invasion of WT Salmonella into WT Hap1 cells expressing the indicated construct: control (-), the SH3 domain of β-PIX (β-PIX-SH3), or the SH3 domain of Abi1 (Abi1-SH3). Invasion values are relative to those in control cells. Invasion of WT Salmonella (20 mins) into MEF (E) or HeLa (F) cells, pretreated with either DMSO, IPA3 or G5555, and also either pretreated with mock or β-PIX siRNA (72 hrs prior to infection). Invasion values are relative to mock siRNA, DMSO treated cells. (G) Salmonella invasion of WT and Δβ-PIX Hap1 cells pretreated with DMSO, IPA3, G5555 or PAK18. Invasion values are relative to those in DMSO treated WT cells. (H) Immunoblot confirming the knockout or knockdown of proteins in the indicated cell lines and conditions. Protein levels quantified from band intensities, relative to total tubulin, are displayed below the immunoblots. All Error bars indicate SD. NS–no significant difference, **—P <0.01, * P <0.05 (ANOVA followed by a post hoc Dunnett’s comparison). (TIF) [file ppat.1009902.s004.tif]

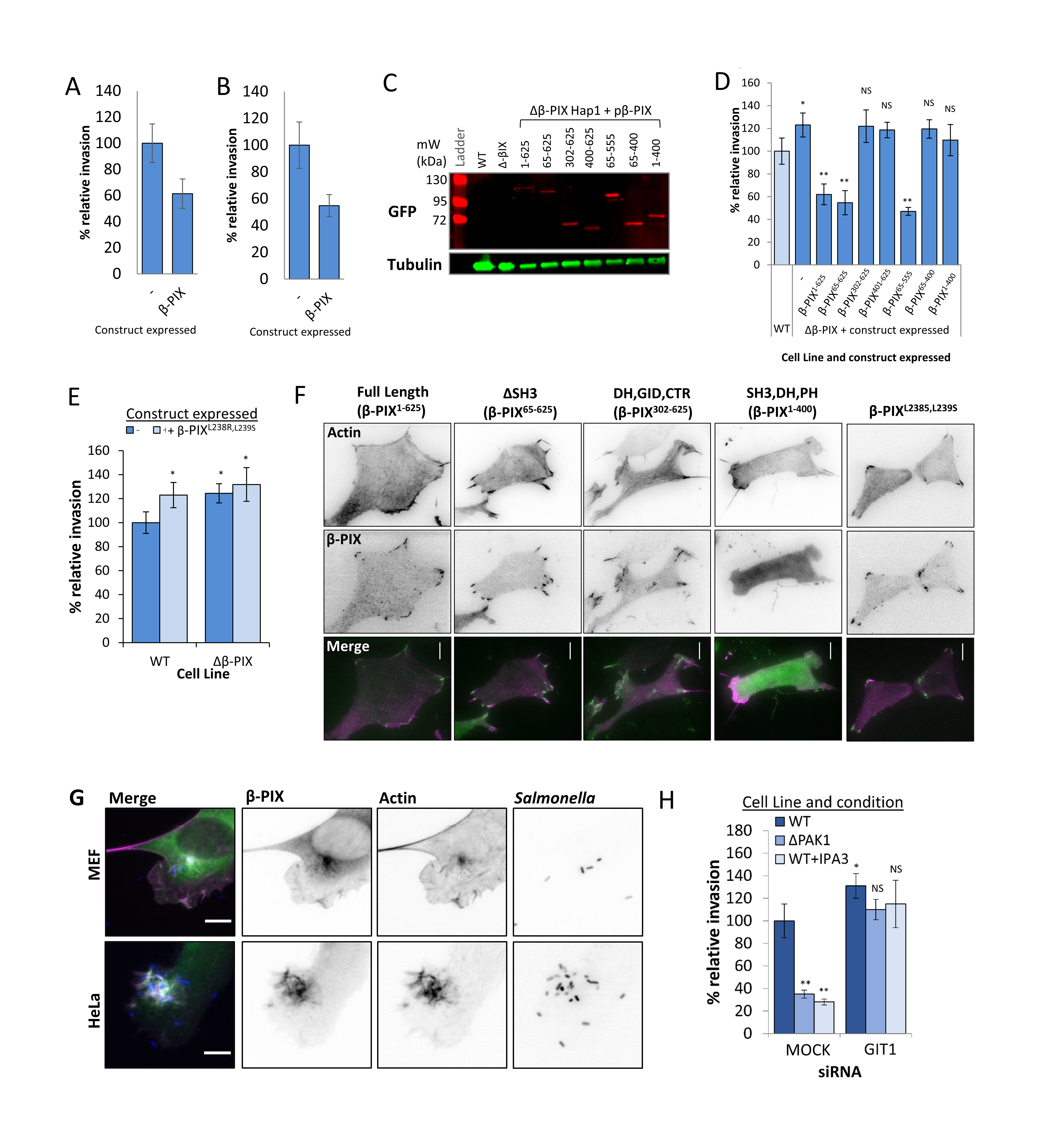

Supplement: S5 Fig — Invasion of WT Salmonella (20 mins) into MEF (A) and HeLa (B) cells expressing no construct (-) or β-PIX, invasion values are relative to corresponding no construct controls. (C) Expression of Full length and truncated β-PIX constructs confirmed by immunoblotting of corresponding cell lysates using a GFP antibody (Red), and a tubulin antibody (Green) as a loading control. (D) Invasion of WT Salmonella (20 mins) into WT and Δβ-PIX Hap1 cells, as well as Δβ-PIX Hap1 cells expressing one the various β-PIX constructs used in (Fig 5A), as indicated. Invasion values are relative to those in WT Hap1 cells. (E) Invasion of WT Salmonella into WT and Δβ-PIX Hap1 cells expressing the indicated construct: control (-) or β-PIX harbouring a mutation in its DH domain that blocks GEF activity (+ β-PIX L238R,L239S). Invasion values are relative to those in control WT Hap1 cells. (F) Fluorescence microscopy images of Δβ-PIX cells expressing indicated Emerald-tagged β-PIX constructs (green). Actin stained with Texas Red Phalloidin (purple). Scale bars are 10 μm. (G) Fluorescence microscopy images of MEF and HeLa cells expressing Emerald-tagged β-PIX (green), infected with Alexa-fluor-350 stained WT Salmonella (10 mins). Actin stained with Texas Red Phalloidin (purple). Scale bars are 10 μm. (H) Invasion of WT Salmonella into WT, ΔPAK1 and WT Hap1 cells treated with IPA3 and pre-treated with either Mock or GIT1 siRNA (72 hrs prior to infection). Invasion values are relative to those in mock siRNA treated WT cells. All Error bars indicate SD. NS–no significant difference, **—P <0.01, * P <0.05 (ANOVA followed by a post hoc Dunnett’s comparison). (TIF) [file ppat.1009902.s005.tif]

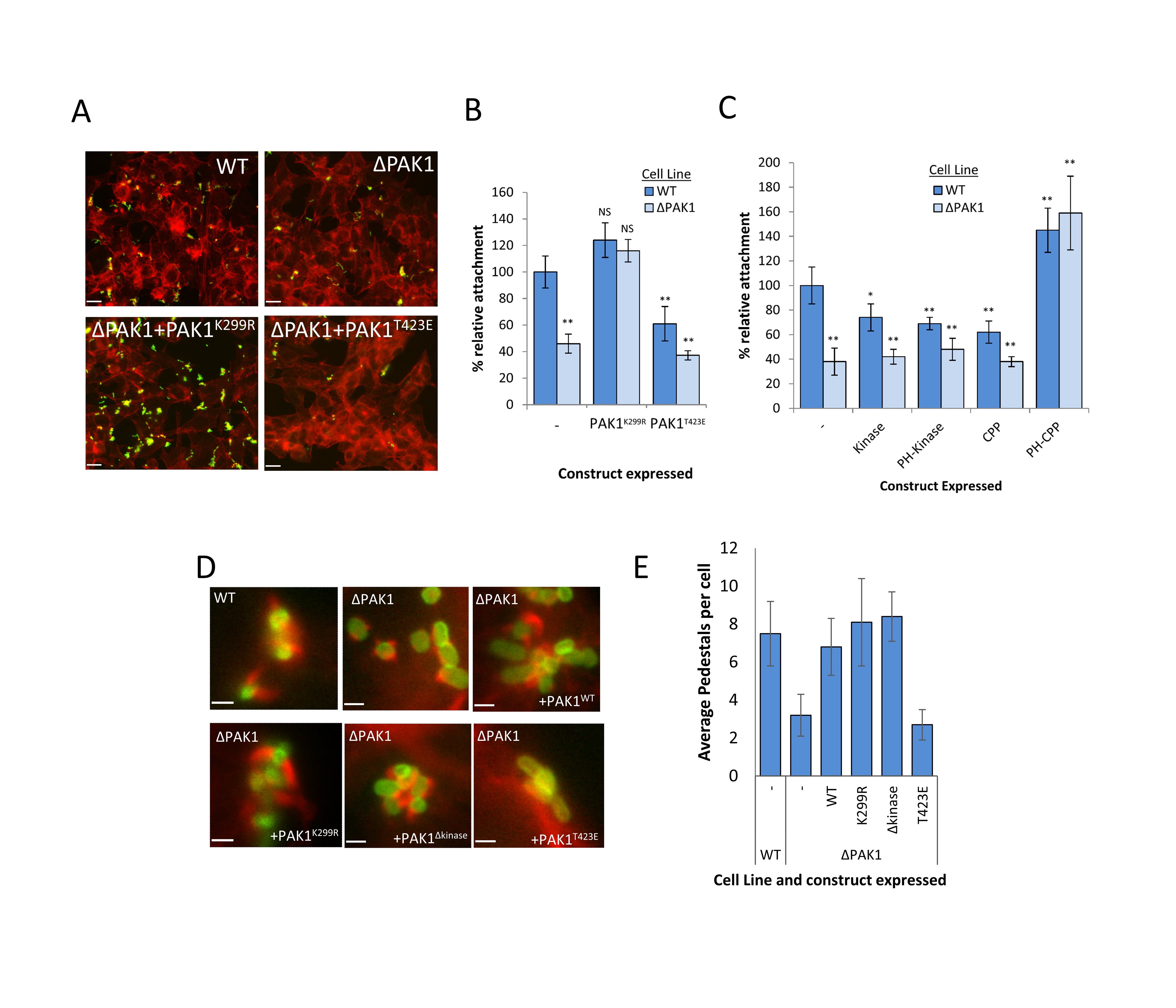

Supplement: S6 Fig — (A) Fluorescence microscopy of EPEC attachment (90 mins) to WT, ΔPAK1 and ΔPAK1 Hap1 cell expressing PAK1K299R or PAK1T423E, cells stained with Texas-red phalloidin to visualise actin (Red) and EPEC stained using an anti-intimin antibody (Green) Scale bar is 20 μm (B) Attachment of WT EPEC to WT and ΔPAK1 Hap1 cells expressing the indicated construct: control (-), kinase-dead PAK1 (PAK1K299R) or constitutively kinase-active PAK1 (PAK1T423E). Attachment values relative to control WT Hap1 cells. (C) Attachment of WT EPEC to WT and ΔPAK1 Hap1 cells expressing the indicated construct: control (-), the kinase domain of PAK1 (kinase), the PAK1 kinase domain fused to the PH domain of ARNO (PH-kinase), the CPP of PAK1 (CPP) or the CPP of PAK1 fused to the PH domain of ARNO (PH-CPP). All attachment values are relative to those in control WT Hap1 cells. (D) Fluorescence microscopy of EPEC pedestal formation (90 mins) to WT, ΔPAK1 and ΔPAK1 Hap1 cell expressing PAK1, PAK1K299R, PAK1Δkinase or PAK1T423E, cells stained with Texas-red phalloidin to visualise actin (Red) and EPEC stained using an anti-intimin antibody (Green). (E) Number of pedestals formed by EPEC per cell (90 mins) on the cells described in (D). All Error bars indicate SD. NS–no significant difference, **—P <0.01, * P <0.05 (ANOVA followed by a post hoc Dunnett’s comparison). (TIF) [file ppat.1009902.s006.tif]

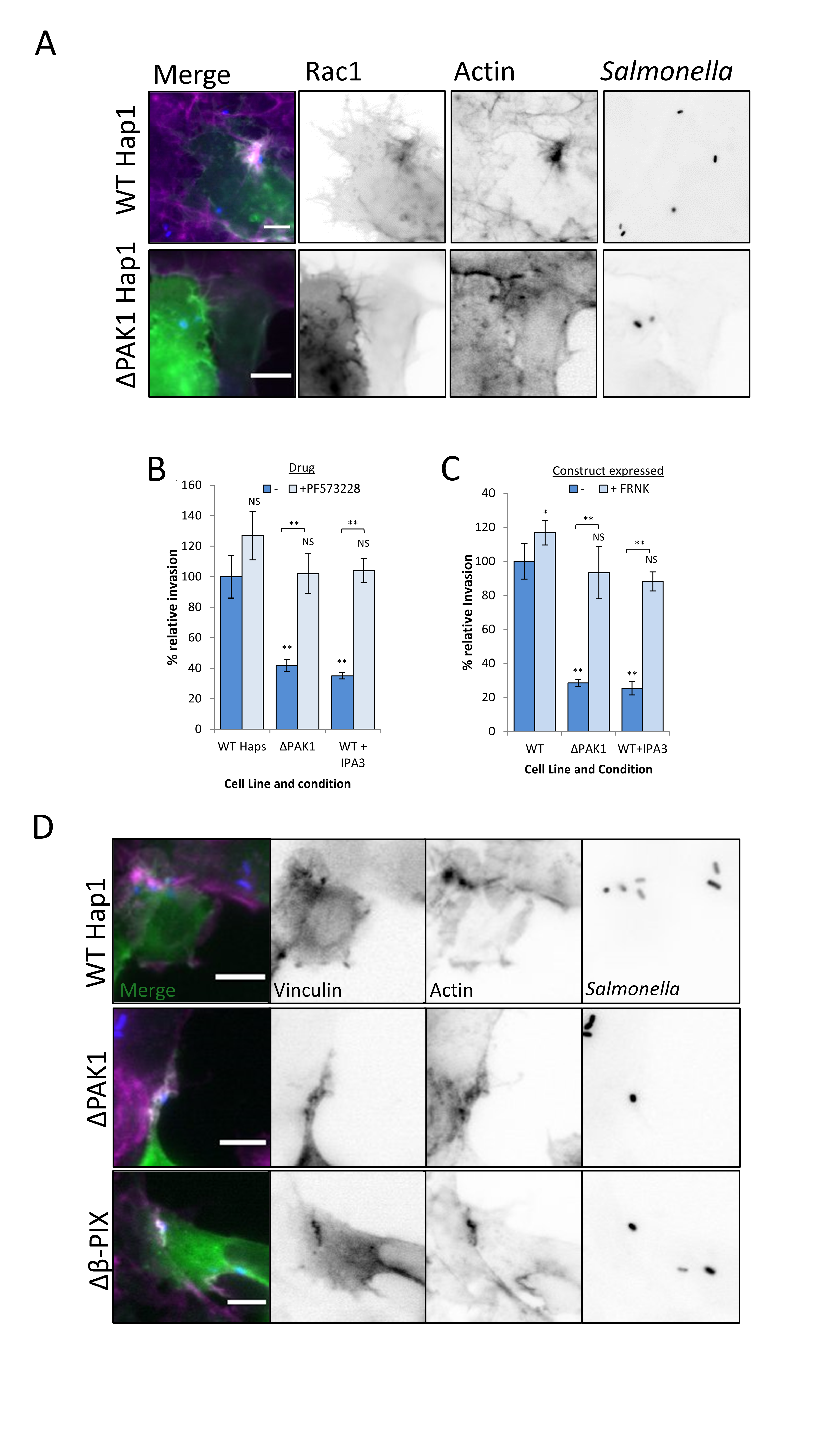

Supplement: S7 Fig — (A) Fluorescence microscopy of WT and ΔPAK1 Hap1 cells expressing Em-Rac1 (green) infected with alexa-fluor 350 stained WT Salmonella (blue– 10 mins). Actin stained with Texas-Red Phalloidin (purple) Scale bars are 10 μm (B) Invasion of WT Salmonella (20 mins) into WT, ΔPAK1 and WT Hap1 cells pretreated with IPA3. Cells were also pretreated with either DMSO (-) or the FAK kinase inhibitor (+PF573228). Invasion values are relative to those in WT control cells. (C) Invasion of WT Salmonella into WT, ΔPAK1 and WT Hap1 cells pretreated with IPA3. Cells expressed indicated constructs: Control (-) or FAK-related nonkinase (+ FRNK). Values are relative to those in control WT Hap1 cells. (D) Fluorescence microscopy of WT, ΔPAK1 and Δβ-PIX Hap1 cells expressing Em-Vinculin (green) infected with alexa-fluor 350 stained WT Salmonella (blue– 10 mins). Actin stained with Texas-Red Phalloidin (purple) Scale bars are 10 μm. All Error bars indicate SD. NS–no significant difference, **—P <0.01, * P <0.05 (ANOVA followed by a post hoc Dunnett’s comparison). (TIF) [file ppat.1009902.s007.tif]
