# Supplementary material for: A kinase-independent function of PAK is crucial for pathogen-mediated actin remodelling
Source: PLoS Pathog. 2021 Aug 30;17(8):e1009902. doi: 10.1371/journal.ppat.1009902 (PMC8432889; doi:10.1371/journal.ppat.1009902)
Supplement: Attachment — Submitted filename: Article Review PLOS Pathogens2021.docx [file ppat.1009902.s009.docx]

*Davidson* *et al.* have presented a study investigating the molecular mechanism of pathogen-driven modulation of host actin cytoskeleton and the role played by PAK. They reveal that when salmonella attach onto host cells, effector proteins stimulate group1 PAK. PAK1 interacts with β-PIX through the central poly-proline rich region (CPP) and inhibits its activity in a kinase independent manner to impede β-PIX activity on cytoskeleton reorganisation. Thus, bacteria and other pathogens modulate host cytoskeleton signalling through PAK1. The paper is well written in general although these are some issues with figure citation as detailed below.

Specific points

The statistical methods were not clearly stated/described and the clarification of using a particular statistical test is also missing. ANOVA should be used for three or more groups of data to find out the relationship or significance between independent and dependent variables.

Figure 1A: WT control could be carried out along with DMSO control to analyse the impact of DMSO in cells.

Figure 1B: Not clear in legend or methods if transfection is transient or stable. This question relates though out the paper – please evidence the level of expression that was achieved in transfection experiments – is it at endogenous levels or overexpression ?

Figure 1E: why was the S144 site selected? What happens at the PAK1 autophosphorylation site in the kinase domain upon pathogen binding? How does this result relate to later discussion of a kinase independent mechanism?

Figure 4D: Cited in text as interaction data but no present in the figure – please check in manuscript citations It would be good to see the Mass Spectroscopy data showing the proteins recruited by the PAK1 polyproline motif.

Figure 5 is not cited in the text but have assumed the first mentions of Figure 6 are actually Figure 5. – please check in manuscript citations

Figure 5D Authors suggest a mechanistic pathway for b-PIX as an inhibitor of Salmonella invasion by Rac1 mis localisation. Please can authors provide Rac1 localisation imaging in WT and Δ PAK1 cells to evidence this hypothesis.

The authors validated the PAK inhibitors experiment in WT MEF and HeLa cells. For knockout, siRNA, overexpression, and localisation experiments used Hap1 cells were used. In the later context it would be important to replicate the results using another cell line. By doing so it would offer the reproducibility of the experiments. This should be mentioned in the discussion.
