# Supplementary material for: A kinase-independent function of PAK is crucial for pathogen-mediated actin remodelling
Source: PLoS Pathog. 2021 Aug 30;17(8):e1009902. doi: 10.1371/journal.ppat.1009902 (PMC8432889; doi:10.1371/journal.ppat.1009902)
Supplement: Attachment — Submitted filename: Response to Reviewers 200721.docx [file ppat.1009902.s010.docx]

Reviewer 1

Major Points

*The authors should clarify how the mutant rescue studies were performed. Aside from one sentence in the Methods, the DNA transfections were not described. Were the knockout cells transiently or stably transfected with the rescue constructs? If transient, how were the transfected cells identified? If stable, how was this accomplished? In either case, were the PAK1 and PIX mutants expressed at the predicted sizes? Ideally, their expression levels would be compared to endogenous PAK1 or PIX.*

The transfections were transient, this is now clarified in the Results and Methods sections. To confirm that constructs were expressed and at the expected size, western blots of cell lysates were probed. We have now included these blots, and they can be found in figures **S2A, S2B, S3A, S4B and S5C**.

*The authors draw several conclusions about the functionalities of PAK (and other) mutants based on fairly modest increases in relative invasion or adherence. However, a few of these conclusions are based on changes of 15-20% with t-test p-values close to 0.05. This is somewhat problematic, because the most appropriate statistical tests for the data depicted in the bar graphs would be ANOVAs (with post-hoc tests). Several conclusions (3ACD, 4DE, 5BC, 6D) might not hold up under these analyses. Which of the increases (e.g., from PAK K299R, PH-CPP, siPIX, and PIX mutant) are confirmed to be statistically significant? If they are not, then several conclusions should be softened.*

This oversight has been corrected and the more appropriate ANOVA followed by post hoc Dunnett's comparison has been utilised to analyse statistical significance. Only three of the data points that were originally considered significant have now changed to being insignificant and graphs have been altered to reflect this. Any mention in the appropriate area of the results section has been adjusted, however none of the changes alter our conclusions. The 3 changes are as follows:

- Figure 5C: βPIX^L283RL239S^ expression in WT Hap1 cells, invasion is no longer significantly greater than invasion in WT Hap1 Control. From * to NS.
- Figure 6D: PH-CPP expression in WT Hap1 cells no longer significantly increases attachment of WT EPEC relative to that in control cells. From * to NS
- Figure S7B: PF573228 treatment of WT Hap1 cells no longer significantly increases invasion compared to that in untreated cells. From * to NS.

*The authors used EPEC to broaden the understanding of how pathogens exploit PAK signaling. But the focus on that part of the manuscript was on bacterial adherence rather than actin rearrangements. To draw the parallels between Salmonella and EPEC, some aspect of F-actin pedestal formation by EPEC should be quantified for the treatments in Fig.6. The acid wash retention measurement is not sufficient in this area. Several representative images analogous to those in Fig.1C, 2D, and S3 should also be incorporated into the main figure or a supplementary figure to support the conclusions.*

We agree and have now included pedestal formation data for some of the key experiments, as well as some representative images of pedestal morphology in certain conditions. These can be found in **Figures 6F, 6G, S6D** and **S6E**. Pedestal formation assay is also described in the methods section.

Minor Issues

*How do the PAK inhibitors work? The peptide inhibitor PAK18 was described, but the mechanisms of actions of the others are unclear. I would’ve guessed that at least one of them targets kinase activity, but the authors’ results suggest that they affect other PAK functions. How do the specific actions of the inhibitors (e.g., IPA3 or G5555) fit with the model that the CRIB-CPP module can bypass their effects during Salmonella signaling?*

We now describe the mechanism of action of all the drugs used in Figure 1 and provide a summary table in **Figure S1A.** All the inhibitors, aside from IPA-3 (which blocks GTPases binding to PAK) directly target ATP binding within in the kinase domain, thereby inhibiting kinase activity.

PAK is autoinhibited and must be phosphorylated (alongside GTPase binding) to adopt and maintain an open, “active” conformation, either via autophosphorylation or by related PAK isoforms (e.g., *Grebeňová et. al, 2019, Scientific Reports*). Our model is that in this open conformation, the CPP domain is exposed and promotes *Salmonella* uptake. Blocking all PAK kinase activity in the cell with chemical inhibitors would therefore block phosphorylation of PAK (we now demonstrate this in Figure S2C) and thus prevent the adoption of the open conformation. Therefore, as the inhibitors work by blocking exposure of CPP, it is expected that expressing the CPP domain alone (e.g. as the PH-CPP fusion) can overcome the block on *Salmonella* invasion.

We agree that this was perhaps not clear and have now explained more fully the role of the PAK phosphorylation and the effect of the chemical inhibitors in both the Results and Discussion sections (Page 4 and 10). In addition, we have altered the title to “ **A** *Kinase independent function of PAK is crucial for pathogen-mediated actin remodelling”* as well as softened our description of the kinase domain as unnecessary to better reflect that whilst kinase activity of some sort is required to activate PAK, it is not required once recruited by *Salmonella*, or indeed EPEC, as this process is driven by the CPP region.

*In addition to the missing Methods regarding transfection/rescue described above, several other aspects of the Methods should be provided, including the concentrations of siRNAs, MOIs for Salmonella- and EPEC-HAP1 infections, and a full section on fluorescence microscopy.*

We have now included siRNA concentrations and MOIs for both *Salmonella* and EPEC in the methods section, we have also included a new fluorescence microscopy section.

*The labels for mutants 83/86 and 107 in Fig.2A appear to be switched.*

We have corrected this error.

Reviewer 2

Major Points

*Introduction: “Previous research has identified a role for PAK in the cell’s
nuclear response to Salmonella infection [20], as well as in the generation of an acute inflammatory
response[21]; however, no role for PAK in bacterial uptake has been demonstrated.” Prior studies by Chen et al (J Exp Med, 1999) showed that kinase-dead mutant of PAK does not affect Salmonella invasion. Proline mutants were also found to have no effect on invasion. This prior work should be cited early on, rather than the discussion.*

We agree and now refer to this in the Introduction.

*The abstract, intro and prior studies by Chen et al (J Exp Med 1999) all indicate PAK kinase activity is not required for Salmonella invasion. So why do PAK kinase inhibitors suppress Salmonella invasion in Figure 1? Same question for Figure 2 data… how can kinase dead PAK1 restore invasion if PAK kinase inhibitors block invasion? Am I missing something?*

We agree that this does seem somewhat confusing, but we feel is entirely consistent with our model. PAK is autoinhibited and must be phosphorylated (alongside GTPase binding) to adopt and maintain an open, “active” conformation, either via autophosphorylation or by related PAK isoforms (e.g. *Grebeňová et. al, 2019, Scientific Reports*). Our model is that in this open conformation, the CPP domain is exposed and promotes *Salmonella* uptake. The kinase inhibitors block all Class I isoforms of PAK in the cell, and therefore block phosphorylation of PAK (we now demonstrate this in Figure S2C), prevent the adoption of the open conformation and also thus inhibit *Salmonella* uptake. The kinase-dead mutant is functional as it is able to be phosphorylated by endogenous PAK isoforms (Figure S2C) in order to expose its CPP domain. This also explains why the inhibitory effects of the kinase inhibitors on *Salmonella* entry can be overcome by expression of the CPP domain, which obviously doesn’t require phosphorylation in order to be exposed.

We agree that this was perhaps not clear and have now explained more fully the role of the PAK phosphorylation and the effect of the chemical inhibitors in both the Results and Discussion sections (Page 4 and 10). In addition, we have altered the title to “ **A** *Kinase independent function of PAK is crucial for pathogen-mediated actin remodelling”* as well as softened our description of the kinase domain as unnecessary to better reflect that whilst kinase activity of some sort is required to activate PAK, it is not required once recruited by *Salmonella*, or indeed EPEC, as this process is driven by the CPP region.

*The authors are using an unconventional invasion assay and need to provide controls, especially considering the fact that Hap1 cells are rarely studied for Salmonella infection. Importantly, are they measuring invasion or rather bacteria associated with the cell surface (i.e. outside)? Does cell surface binding change with altered PAK activity/expression?*

We apologise for not making this clearer. The method used to measure invasion only looks at internalised bacteria. The *Salmonella* carry a plasmid (pM975 - which was developed in the lab of Wolf-Dietrich Hardt) that only expresses GFP in response to an environment with a low pH (i.e. when internalised in *Salmonella-*containing vesicles). Other than this, the assay is a standard gentamycin protection assay in which all external bacteria are killed, leaving only those that have entered the cell. This assay has been utilised in numerous research articles such as ***(****Misselwitz et. al, 2011, Infection and Immunity),* [*(Vishwakarma*](https://www.ncbi.nlm.nih.gov/pubmed/?term=Vishwakarma%20V%5BAuthor%5D&cauthor=true&cauthor_uid=22753379) *et. al, 2012, Infection and Immunity), (Sellin et. al, 2014, Cell host & Microbe****)*** as well as several of our own publications. We have made the description of this assay clearer in the Methods section.

*Where is the mass spectrometry data for His-PSB-CPP vs His-PSB alone? This is discussed in text but not shown anywhere.*

The raw mass spectrometry data has now been included as a supplemental file and is mentioned on page 6 of the manuscript.

*-Figure 4. The interaction of PH-CPP construct with b-PIX is shown. What about full length PAK1… does it bind as well? A co-ip assay should be attempted. Does binding change during infection?*

We have performed a GFP-Trap using cells expressing Emerald tagged PAK1 and can confirm that full-length PAK is able to interact with β-PIX and GIT1 **(Figure 5D).** During *Salmonella* infection, whilst we see an increase in the levels of active PAK (P-PAK1^ser144^) there does not seem to be a significant increase in the amount β-PIX bound to PAK. This might suggest that instead of recruiting β-PIX during infection, that the whole PAK1-β-PIX-GIT complex becomes newly localised to *Salmonella* invasion foci, instead of in local focal adhesions. This is discussed in the results section on page 7.

*Is b-PIX affecting recruitment of FA markers to Salmonella invasion sites?*

We have now examined the localisation of transiently over-expressed fluorescently-tagged vinculin (Figure S7D). Whilst we do see accumulation of vinculin around the invasion site, it is difficult to observe any obvious changes in either ∆PAK1 or ∆β-PIX cells, other than when a ruffle fails to form and no vinculin is recruited, as might be expected. This is described in the discussion section (Page 11).

*Authors indicate they are examining “attachment” – are they referring to EPEC pedestals (A/E lesion formation) or just passive binding to the cell surface? Images are required to show what they are measuring.*

For the sake of space, we only included attachment data, which we describe as EPEC that are still tightly bound to the cell after extensive washing. We now show example images of what we are measuring in **Figure S6A**. We have now also included some pedestal data for key experiments (both representative images and quantitation of pedestal numbers) in **Figure 6F, 6G, S6D and S6E.**

Minor Points

*Figure S2: The wild type PAK1 control should be added to this figure.*

The WT PAK1 recruitment image can be found in **figure 1C.**

*Figure 3B would benefit from a column indicating “Restores invasion” similar to Figure 2B*

This column has been added to **figure 2B.**

Reviewer 3

Major Points

*The statistical methods were not clearly stated/described and the clarification of using a particular statistical test is also missing. ANOVA should be used for three or more groups of data to find out the relationship or significance between independent and dependent variables.*

We have corrected this oversight and performed the more appropriate ANOVA followed by post hoc Dunnett's comparison. Three of the data points that were originally considered significant have now changed to being insignificant. Changes in the appropriate part of the results section have been made, but these changes do not affect any of the conclusions. The following changes were made:

- Figure 5C: βPIX^L283RL239S^ expression in WT Hap1 cells, invasion is no longer significantly greater than invasion in WT Hap1 Control. From * to NS.
- Figure 6D: PH-CPP expression in WT Hap1 cells no longer significantly increases attachment of WT EPEC relative to that in control cells. From * to NS
- Figure S7B: PF573228 treatment of WT Hap1 cells no longer significantly increases invasion compared to that in untreated cells. From * to NS.

*Figure 1A: WT control could be carried out along with DMSO control to analyse the impact of DMSO in cells.*

Untreated control has been added to Figure 1A, DMSO has no significant impact on the cells relative to this.

*Figure 1B: Not clear in legend or methods if transfection is transient or stable. This question relates though out the paper – please evidence the level of expression that was achieved in transfection experiments – is it at endogenous levels or overexpression ?*

Throughout the results section, and in the methods section, we make clear that the constructs were transiently over-expressed. Western blots were used to confirm that all constructs were overexpressed and were the predicted sizes **(Figures S2A, S2B, S3A, S4B and S5C).**

*Figure 1E: why was the S144 site selected? What happens at the PAK1 autophosphorylation site in the kinase domain upon pathogen binding? How does this result relate to later discussion of a kinase independent mechanism?*

Upon activation, PAK adopts an 'open' active conformation, which is stabilised by autophosphorylation of various residues, including Ser144. The level of phosphorylation at Ser144 is therefore a readout for the formation of the 'open' active state. We did also attempt to probe phosphorylation at Thr423 (within the kinase domain) but were unable to find an antibody that gave consistent results in our hands.

We refer to a kinase-independent mechanism as it is the CPP domain of PAK that is responsible for the signalling that drives *Salmonella* entry. However, this domain is only exposed when PAK is in the open active conformation induced (alongside GTPase binding) by Ser144 phosphorylation - either intra/intermolecular autophosphorylation or via closely-related Pak isoforms (this is why PAK kinase inhibitors impair *Salmonella* entry even though it is "kinase-independent"). Whether Thr423 is phosphorylated or not upon *Salmonella* entry would not affect our model in any way. We have however now clarified what we mean by a "kinase-independent" function of PAK, better described our model for what is happening and include discussion of how kinase activity may play a role in Ser144 phosphorylation and stabilising of the open active conformation (Pages 3, 4 (results section) and 9 (discussion)).

*Figure 4D: Cited in text as interaction data but no present in the figure – please check in manuscript citations It would be good to see the Mass Spectroscopy data showing the proteins recruited by the PAK1 polyproline motif.*

The raw mass spectrometry data is now available as a supplemental file and is referred to in the in the results section (Page 6).

*Figure 5 is not cited in the text but have assumed the first mentions of Figure 6 are actually Figure 5. – please check in manuscript citations*

This was a mistake and we have now changed all references to Figure 6 to Figure 5.

*Figure 5D Authors suggest a mechanistic pathway for b-PIX as an inhibitor of Salmonella invasion by Rac1 mis localisation. Please can authors provide Rac1 localisation imaging in WT and Δ PAK1 cells to evidence this hypothesis.*

We have now provided images of Rac1 recruitment by *Salmonella* in WT and ∆PAK1 Hap1 cells (Figure S7A). Whilst Rac1 is efficiently recruited into the membrane ruffle in WT cells, the vast majority of *Salmonella* do not form extensive ruffles in ∆PAK1 cells, though it is still possible to see some small amount of Rac1 recruitment. Whether poorer Rac1 recruitment is the reason for the ruffle not forming (and subsequently poor invasion), or whether Rac1 is not well recruited due to the absence of a ruffle is difficult to ascertain here. We leave the possibility that β-PIX interferes with Rac1 localisation as an open question and describe this in the discussion.

*The authors validated the PAK inhibitors experiment in WT MEF and HeLa cells. For knockout, siRNA, overexpression, and localisation experiments used Hap1 cells were used. In the later context it would be important to replicate the results using another cell line. By doing so it would offer the reproducibility of the experiments. This should be mentioned in the discussion.*

We agree that up until now Hap1 cells have not frequently used to investigate *Salmonella* invasion, though they have been very useful for us in looking at knockout and complementation. To help validate our results from the Hap1 cells we have performed numerous extra key experiments (that do not require knockout cell lines) in both MEF and HeLa cells, including those using siRNA as well as PAK and β-PIX over-expressions. These extra experiments, that backup our existing data can now be found in Figures S1E, S2B, S2C, S3E, S3F, S4A, S4B and S4G.
